# Diode-Laser-Based Raman Spectroscopy Applied to the Thermodynamic Characterization of Natural Gas and Hydrogen-Enriched Natural Gas

**DOI:** 10.3390/s26123820

**Published:** 2026-06-16

**Authors:** Fabio Melison, Lorenzo Cocola, Elena Meneghin, Riccardo Danese, Daniele Rossi, Luca Poletto

**Affiliations:** 1 CNR-IFN, National Research Council of Italy—Institute for Photonics and Nanotechnologies, Via Trasea 7, 35131 Padova, Italy; lorenzo.cocola@cnr.it (L.C.); luca.poletto@cnr.it (L.P.); 2 Pietro Fiorentini S.p.A., Via Enrico Fermi 8/10, 36057 Arcugnano, Italy; elena.meneghin@fiorentini.com (E.M.); riccardo.danese@fiorentini.com (R.D.); daniele.rossi@fiorentini.com (D.R.)

**Keywords:** Raman spectroscopy, natural gas, hydrogen-enriched natural gas, higher heating value, in-line gas analysis, industrial-grade spectrometer, hydrogen blending

## Abstract

**Highlights:**

**What are the main findings?**

- A Raman-based instrument was developed for in-line compositional analysis of natural gas and hydrogen-enriched natural gas, enabling continuous calculation of gas-quality parameters such as higher heating value.
- The system was validated under industrially relevant conditions, achieving OIML R 140 Class A performance over a wide pressure and temperature range without carrier gases or sample manipulation.

**What are the implications of the main findings?**

- The results demonstrate that Raman spectroscopy can provide a robust and low-maintenance alternative for distributed natural-gas-quality monitoring in transportation and distribution networks.
- The instrument supports real-time monitoring of hydrogen blending and variable gas compositions, contributing to safer and more flexible operation of future gas infrastructures.

**Abstract:**

Natural gas transportation and distribution networks are becoming increasingly heterogeneous due to the injection of biomethane, regasified LNG, and hydrogen-enriched natural gas, requiring distributed and continuous gas-quality monitoring. This work presents an industrial Raman-based instrument for in-line measurement of natural gas and hydrogen-enriched natural gas composition and related thermodynamic properties. The system employs a 450 nm broadband laser diode, a high-throughput custom spectrometer, and a pressure-rated gas cell integrated in an ATEX-certified enclosure. Gas composition is retrieved through calibration spectra and non-linear least-squares fitting, while higher heating value is calculated according to ISO 6976. The instrument was validated over pressures from 1.5 to 17 bara and temperatures from −20 °C to 55 °C using certified representative gas mixtures. The system achieved compliance with OIML R 140 Class A requirements, with HHV errors below ±0.5% and repeatability within 0.1%, while operating without carrier gases or sample manipulation. Long-term field operations in pressure-reduction stations confirmed stable performance over twelve months. The results demonstrate that Raman spectroscopy can provide a robust, low-maintenance solution for continuous natural-gas-quality monitoring and controlled hydrogen-blending applications.

## 1. Introduction

The continuous monitoring of the chemical composition of natural gas (NG) is a crucial aspect of both energy distribution and safety management in modern infrastructures. Nowadays, various techniques are employed to measure NG, each relying on different approaches depending on the specific motivation behind the measurement. The main reasons can be summarized as follows: (i) Economic and commercial purposes: NG is traded on both volumetric and energetic bases, as the amount consumed for a specific application is strictly related to the gas quality and therefore to the energy that can be extracted from its combustion. (ii) Quality control: NG is not a pure substance but a mixture mainly composed of methane. Its composition strongly depends on its origin and on any blending process. (iii) Network safety and integrity: Continuous monitoring allows for the prevention and early detection of corrosion or material degradation.

In addition, environmental monitoring and leak detection represent complementary applications in NG infrastructure management. Unlike compositional analysis, these approaches are generally focused on detecting methane or combustible-gas releases, rather than determining the complete chemical composition of the gas mixture [1,2].

In recent years, the analysis of NG composition and the calculation of its thermodynamic properties have become increasingly crucial due to the growing diversification of NG sources. Historically, the transportation and distribution networks resembled a largely unidirectional flow, supplied by a limited number of sources and distributed with minimal interaction; it is now evolving into a more distributed and heterogeneous system. Within this network, characterized by a rising number of injection and blending points, storage facilities act as backups during periods of peak consumption. The use of regasified Liquefied Natural Gas (LNG) is also becoming more widespread. The local injection of biomethane, produced via biogas upgrading and largely comparable to pure methane, introduces further variability. Although nearly pure methane, biomethane can alter the thermodynamic properties of the gas mixtures flowing through the network. Hydrogen, increasingly employed as a green energy carrier, is also attracting attention for blending with NG to form hydrogen-enriched natural gas (HNG), further contributing to the heterogeneity of the network [3,4,5,6,7,8]. Given this evolution, national regulatory authorities no longer accept sampling solely at entry and distribution points. Instead, widespread sampling across the network is required, particularly in areas near blending and injection sites, such as regasification terminals or biomethane production facilities. These samplings are primarily used for economic accounting between national system operators and local distributors, while also preventing the formation of gas mixtures with non-compliant energy content within the network.

The quality of a combustible gas mixture is primarily characterized by its higher heating value (HHV), defined as the total energy released during complete combustion, including the latent heat of water condensation. The HHV is calculated from the molar fractions of the mixture components weighted by their individual calorific values (Equation (1)).

(1) $${HHV}_{mix}=\sum_{i=1}^{N}\left(x_{i}*{HHV}_{i}\right),$$

In Equation (1), *x_i_* represents the molar fraction of the *i-th* component and *HHV_i_* its corresponding calorific value on molar basis. The HHV can also be expressed on mass or volumetric basis. The ISO 6976:2016 [9] allows the thermodynamic properties of real, not only ideal, gases to be determined based on their composition. This standard defines the calculation procedure for the HHV, as well as related thermodynamic properties such as molar mass, Wobbe index, absolute and relative density and compressibility factor Z [9]. Table 1 reports the HHV of the main NG components and hydrogen.

The composition of NG varies significantly: methane typically ranges from 80% to 100%, ethane up to 10%, propane up to 5%, and butanes up to 2%, while N_2_ and CO_2_ are usually below 3%. Heavier hydrocarbons and other species are present in trace amounts. Hydrogen content may range from 0% to 20%, depending on application and regulatory constraints.

Mixtures with higher concentrations of heavy hydrocarbons exhibit increased HHV, whereas non-combustible species such as N_2_ and CO_2_ reduce the overall energy content. Although hydrogen is combustible, its lower HHV compared to methane results in a net decrease when blended into NG. These dependencies highlight the need for accurate compositional analysis to reliably determine HHV, particularly in increasingly heterogeneous gas networks and for compliance with operational and regulatory requirements [10,11].

## 2. Natural Gas Analyzers: State of the Art

Gas Chromatography (GC) is the reference method for determining NG composition and deriving thermodynamic properties. Miniaturized GC systems (mini-GCs) enable field deployment by directly sampling gas from the network and performing separation using carrier gases (e.g., He or Ar) and chromatographic columns, with detection via a Thermal Conductivity Detector (TCD). Their compact design allows reduced analysis times (2–10 min), although at the expense of separation efficiency in complex mixtures. GC remains the established reference technique also for HNG analysis. The literature on the subject is extensive and widely available, and the main international standards explicitly refer to this specific technique [10,11,12,13,14]. In industrial NG applications, mini-GCs typically achieve LODs of 10–100 ppm for hydrocarbons, N_2_, and CO_2_, and around 100 ppm for H_2_ with a dedicated column and the use of helium as carrier gas. Their accuracy and repeatability are generally better than ±0.5% of the molar fraction [15,16].

Mini-GCs present inherent limitations, including the need for a continuous carrier gas supply and associated cylinders, as well as detector drift during prolonged operation, requiring periodic recalibration with reference gases. Additionally, their high capital and maintenance costs limit widespread deployment to strategic locations across NG networks.

Electrochemical sensors and MEMS represent other contact-based approaches for gas analysis. Electrochemical sensors rely on redox reactions to detect target species with high sensitivity; however, they suffer from long-term drift, cross-sensitivity, hysteresis, and non-linearity, particularly in complex mixtures. As a result, they are mainly used for leak detection rather than accurate compositional analysis in NG applications [17,18,19,20]. MEMS-based sensors exploit variations in thermal conductivity or resonance frequency to detect gas concentrations, offering compactness, low power consumption, and potentially high sensitivity. However, in NG applications, these devices are generally more suitable for indirect calorific-value estimation than for detailed compositional analysis. Although legal-metrology-compliant instruments based on these principles exist, their limited molecular selectivity and sensitivity to gas-matrix variations, contaminants, and environmental conditions can limit their applicability in applications requiring high analytical accuracy [21,22]. Correlative industrial calorimeters have also been developed for natural-gas-quality monitoring. For example, temperature-dependent thermal-conductivity measurements combined with support vector regression have been used to estimate calorific value, Wobbe index, methane number, and density in natural gas calorimeters. Nevertheless, compared with analytical systems designed for fiscal metering, these instruments generally provide a lower accuracy class and are therefore more suited to process monitoring than to high-accuracy fiscal applications [23].

Tunable Diode Laser Absorption Spectroscopy (TDLAS) and Fourier Transform Infrared (FTIR) spectroscopy are absorption-based techniques exploiting molecular transitions at characteristic photon energies. TDLAS provides high sensitivity (ppm–ppb) and selectivity, but requires multiple laser sources for multi-species analysis, increasing system complexity and cost. FTIR enables broadband, simultaneous detection and performs well for hydrocarbons and CO_2_; however, both techniques are limited in NG applications by the weak or absent IR activity of homopolar molecules. In particular, N_2_ shows no IR-active transitions, while H_2_ exhibits only weak absorption lines at 2121.8 nm and 4712.9 nm, limiting its detectability [24,25]. Although TDLAS has been demonstrated for hydrogen detection, the operating conditions of these experimental setups are not compatible with industrial NG requirements. Advanced variants of TDLAS and FTIR have also been investigated for gas mixture analysis. Cavity-Enhanced and Cavity Ring-Down Spectroscopy (CEAS/CRDS) improve detection sensitivity by increasing the effective optical path length [26], while step-scan and time-resolved FTIR enhances spectral resolution and temporal response [27]. However, these techniques remain sensitive to mechanical and environmental instabilities and do not overcome the intrinsic limitations in detecting homopolar molecules.

Raman spectroscopy probes molecular vibrational (and rotational, in gases) energy levels through inelastic scattering of incident photons, providing species-specific spectral fingerprints. The Raman signal intensity depends on the excitation wavelength (∝1/λ^4^), optical power, molecular cross section, and sample density [28,29,30,31,32]. The choice of excitation source therefore represents a trade-off between efficiency, cost, fluorescence effects, and optical design constraints. Raman spectroscopy is well established for solid and liquid analysis, but its application to gases is limited by the intrinsically low scattering efficiency and the strong dependence on sample density, resulting in significantly weaker signals. Consequently, gas-phase Raman instrumentation has been extensively investigated, but its implementation in industrial applications remains relatively limited. Several studies have demonstrated the capability of Raman spectroscopy to accurately analyze NG mixtures under controlled conditions, often through optimized laboratory setups. However, these approaches typically rely on laboratory-based instrumentation, such as cooled detectors and high-resolution spectrometers, which are not compatible with industrial constraints in terms of robustness and cost [33,34,35,36]. In addition, signal-enhancement strategies such as cavity-enhanced and multi-pass Raman configurations have been investigated to overcome the intrinsically weak Raman response of gases and push detection limits under optimized laboratory conditions [37,38]. In contrast, the present work does not aim at maximizing trace-gas sensitivity through optical enhancement schemes, but at implementing a compact, robust, and field-deployable Raman analyzer for NG and HNG gas-quality monitoring under industrial operating conditions. This work presents a Raman-based system for NG and HNG analysis specifically designed to meet industrial and metrological requirements.

## 3. Materials and Methods

The design and development of the system presented in this work were guided from the outset by the typical requirements of industrial environments. Particular attention was devoted to the system’s power consumption, robustness, and the adoption of a simple and modular implementation. For this reason, a modular and streamlined architecture was selected to facilitate both assembly and subsequent optical alignment. As many commercially available components as possible were employed; no custom optics were used, and all parts can be sourced from standard suppliers.

Due to the intrinsically weak Raman signal in gases, a high-throughput diffraction-grating spectrometer was specifically developed and integrated into the instrument. The required operating temperature range (−20 °C to 50 °C) precludes the use of conventional 532 nm DPSS lasers, which lack operativity under such conditions or require prohibitively expensive ruggedized solutions. A continuous-wave high-power semiconductor laser diode with 450 nm central wavelength and 5 W maximum power was selected as excitation source due to its high efficiency, low cost, compactness, and robustness. The 450 nm central wavelength enhances Raman scattering efficiency compared to standard 532 nm excitation by almost a factor of 2. However, the source exhibits poor beam quality, with high divergence (9° × 49°), multimode emission, and large M^2^, resulting in a non-circular beam profile and astigmatism. Although the poor beam quality could introduce spatially induced spectral broadening, the dominant contribution arises from the intrinsic laser linewidth (FWHM ≈ 2 nm), which largely masks beam-related effects. The emission wavelength and optical power of the laser diode are temperature-dependent; therefore, a Thermo-Electric Cooler (TEC) is employed to stabilize the diode at 35 °C, ensuring consistency between calibration and measurement spectra.

The novelty of the proposed system does not rely solely on the use of a broadband diode laser as the excitation source, but rather on the overall instrumental architecture, which was developed from the outset with industrial deployment as a primary design constraint. The system combines a broadband diode laser, a custom high-throughput spectrometer, an uncooled industrial CMOS camera, a pressure-rated gas cell, and an ATEX-certified enclosure. The design and integration of these components were specifically aimed at enabling NG and HNG measurements outside controlled laboratory environments, where the instrument is exposed to wide temperature ranges, variable operating pressures, and changing gas compositions according to gas-network conditions, without requiring sample handling or carrier gases.

The use of a broadband diode laser should therefore be regarded as a deliberate engineering trade-off rather than as a strategy to maximize trace-species sensitivity. The 450 nm excitation wavelength increases the Raman scattering efficiency compared with conventional 532 nm sources, while the diode architecture improves compactness, cost effectiveness, and robustness. However, the intrinsic linewidth of the source broadens the recorded Raman features. For this reason, the present instrument is designed as an industrial gas-quality analyzer for the main NG and HNG components and HHV determination, rather than as a trace-contaminant analyzer.

### 3.1. Opto-Mechanical Realization

**Figure 1:**
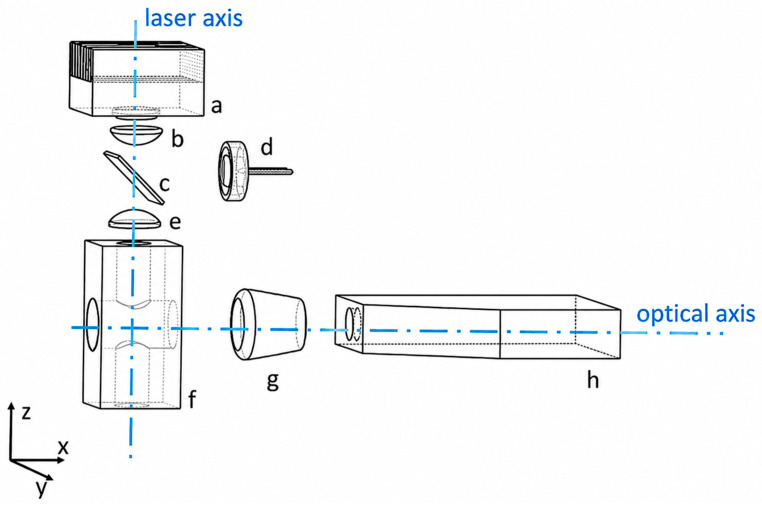
Opto-mechanical layout of the system: (a) laser source section, (b) collimation lens, (c) beam splitter, (d) photodiode, (e) focusing lens, (f) gas cell, (g) coupling optics, (h) spectrometer.

The instrument adopts a 90° geometry in which the laser axis forms a 90° angle with the optical axis of the spectrometer [39,40]. A schematic overview of the instrument is reported in Figure 1.

From an opto-mechanical standpoint, the system consists of the functional parts labeled in Figure 1:

(a) Laser source section. The laser diode is mounted in a custom designed TEC module (Copper housing). The beam propagates along the laser axis with polarization orthogonal to the optical axis, ensuring stable coupling into the optical system. The TEC stabilizes the temperature at 35 °C and drives the diode at 1.5 A, providing about 2 W optical power.
(b) Collimation lens. Due to the high beam divergence and small emitter size, a ½″ diameter, 20 mm focal length lens is mounted directly on the TEC housing to efficiently collimate the laser beam.
(c) Beam splitter. Positioned in the collimated beam path, it splits ~4% of the optical power toward a photodiode for real-time source monitoring.
(d) Photodiode. The deflected beam fraction is measured by a photodiode to provide real-time laser-power monitoring for spectral normalization, as well as diagnostics for possible source degradation.
(e) Focusing lens. A 1″ diameter, 50 mm focal length lens focuses the beam at the center of the gas cell, generating a ~150 µm waist and an interaction length of ~8 mm for Raman scattering.
(f) Gas cell. This is the only component in contact with the sample and is designed for operation up to 17 absolute bar (bara), enabling direct integration into NG networks without depressurization. 1″ diameter broadband-coated optical windows allow Raman signal collection while minimizing stray light. The transmitted beam is terminated in a built-in beam dump integrated into the gas outlet; the adopted design improves robustness and eliminates additional optical components [41]. Before entering the cell, the gas sample passes through porous filters to remove suspended particulate matter that could generate strong spurious signals.
(g) Coupling optics. The laser–gas interaction region is imaged onto the entrance plane of the spectrometer by two lenses giving a 2× demagnification factor and f/3.2 aperture; that is the effective collecting angle of the overall system.
(h) Spectrometer. The Raman spectrum is acquired by a lens-based Czerny–Turner spectrometer, featuring an effective f/2.0 aperture, a 460 nm long-pass filter to block the intense Rayleigh scattering from the fundamental, a 1200 grooves/mm diffraction grating giving an average spectral dispersion of 20 nm/mm. The spectrum is finally acquired by an uncooled CMOS sensor (1936 × 1216 pixels, 5.86 µm pitch). This configuration enables detection from the filter cut-off to beyond the H_2_ Raman line at 4156 cm^−1^.

**Figure 2:**
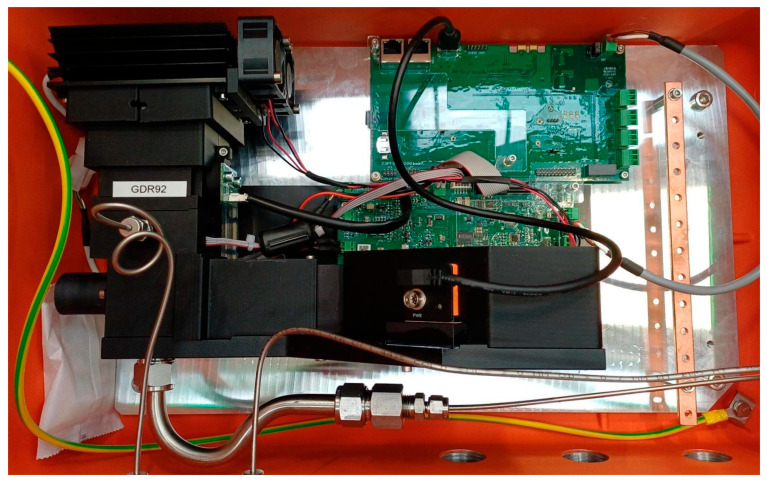
Picture of the Raman system allocated inside an ATEX box.

A picture of the instrument is shown in Figure 2. The full system is housed in an ATEX-certified explosion-proof enclosure for gas group IIB + H_2_, suitable for Zone 1 operation and selected according to its ATEX certification and the applicable requirements of the IEC 60079 series [42]. The integrated control electronics leave only the external interfaces required for operation accessible, namely the gas inlet and outlet, electrical power, and data connections. The total power consumption is below 30 W.

### 3.2. Data Acquisition and Spectral Processing

**Figure 3:**
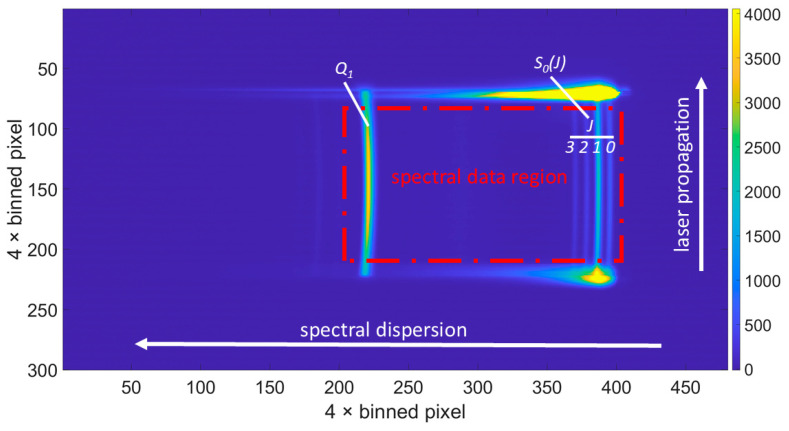
Hydrogen Raman emission recorded by the system, 40 s integration time, 2 bar gas pressure. The intense feature labeled Q_1_ corresponds to the H_2_ vibrational Raman Q-branch, centered at approximately 4155 cm^−1^. The four features labeled S_0_(J) correspond to the pure rotational Raman transitions of H_2_ close to the excitation wavelength, i.e., S_0_(0), S_0_(1), S0(2) and S0(3) occurring at 354 cm^−1^ and 587 cm^−1^, 814 cm^−1^ and 1035 cm^−1^, respectively.

The analytical workflow consists of a one-time laboratory calibration followed by climatic and in situ measurements. The acquisition sequence includes laser thermalization, dark-frame acquisition, laser activation, and spectral collection with corresponding photodiode signals, followed by laser shutdown. During the calibration procedure the gas cell pressure is also acquired. An acquired spectrum is shown in Figure 3 for pure hydrogen Raman emission.

In Figure 3, the vertical axis corresponds to the laser propagation direction, while the horizontal axis represents spectral dispersion. The selected region of interest defines the usable data, limited vertically to the length of the laser–gas interaction region (~8 mm). Field curvature due to Petzval aberration is observed along the vertical axis [43] and is software corrected using a first-order software approach similar to that described in [44]. The horizontal displacement of an intense Raman feature was estimated along the vertical detector coordinate, and each detector row was shifted with respect to the center row. The same correction was applied to both calibration and measurement spectra. This first-order correction is adequate for the present application, where the recorded gas Raman bands are mainly broadened by the intrinsic linewidth of the diode laser. Spectra are finally obtained summing the signal along the laser-propagation axis.

**Figure 4:**
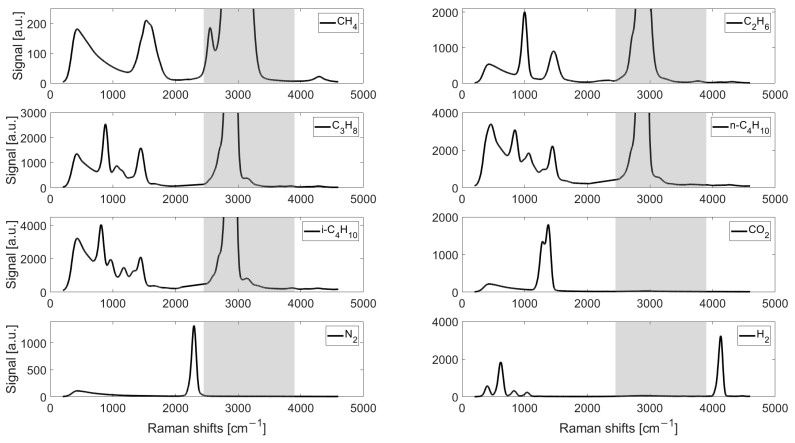
Raman calibration spectra, 60 s integration time.

Calibration spectra for each NG and HNG component (reported in Table 1) were acquired using certified gas mixtures at known pressures, which have been monitored by a precision pressure gauge with 0.05% full scale accuracy. Figure 4 shows the calibration spectra, normalized for integration time, laser power, gas pressure, and concentration.

A software correction was applied when binary mixtures were used during calibration. This was the case for butanes: due to their low liquefaction pressure, approximately 2 bar at 20 °C, their calibration spectra were obtained from binary mixtures in nitrogen. The nitrogen contribution was removed using the nitrogen calibration spectrum itself. Since its Raman emission was known, it was possible to subtract its contribution by applying a software correction to the hydrocarbon spectrum acquired in binary mixture with nitrogen. Additionally, the spectral region around 2914 cm^−1^ was excluded from further processing because it is dominated by the non-selective C–H stretching band, which does not provide component-specific information for hydrocarbons with the specific setup used in this study. As can be observed in Figure 4, the combination of the spectral linewidth of the excitation source and the dispersion of the spectrometer makes this region unsuitable for selective analysis. These processing steps are performed only once during the calibration phase and are not intended to selectively remove spectral discrepancies due to unknown components. Once the calibration set has been generated, each measured NG and HNG spectrum is elaborated using only these spectral contributions. The region around 2914 cm^−1^ was excluded both during calibration and during analysis. This approach improves the robustness of the fitting database by preventing known non-target contributions from being associated with calibration spectra. Nevertheless, it does not remove the intrinsic limitations associated with overlapping Raman bands. If non-calibrated species are present in the sample, they may increase the fitting residuals and introduce bias in the retrieved concentrations.

The calibration spectra exhibit significant broadening, with features such as the CO_2_ doublet appearing merged and the H_2_ Raman line (4156 cm^−1^) showing a FWHM of ~80 cm^−1^. This effect is dominated by the intrinsic linewidth of the laser diode, exceeding pressure-induced and temperature-induced spectral broadening. Consequently, the calibration spectra remain valid across the full operating pressure range (1.1–17 bara), as instrumental broadening masks pressure-dependent effects.

After calibration, NG and HNG measurements are performed using the same spectral processing routine, excluding pressure normalization. The mixture composition is retrieved by fitting each experimental spectrum with a linear combination of calibration spectra using a Levenberg–Marquardt non-linear least-squares algorithm. The fitting coefficients *α_i_* represent the contributions of each component and are constrained to be non-negative (Equation (2)).

(2) $$\underset{\alpha_{1},\alpha_{2},\ldots ,\alpha_{N}}{\min}\left.|s_{\exp}-s_{\mathrm{synth}}\right.{|}^{2}=\underset{\alpha_{1},\alpha_{2},\ldots ,\alpha_{N}}{\min}\left.|s_{\exp}-\left(\alpha_{1}\;{\mathrm{cal}}_{1}+\alpha_{2}\;{\mathrm{cal}}_{2}+\cdots +\alpha_{N}\;{\mathrm{cal}}_{N}\right)\right.{|}^{2},$$

Due to the applied normalization, the fitting coefficients directly correspond to the partial pressures of the mixture components [36,39], enabling estimation of both total pressure and volumetric composition. Thermodynamic properties are then calculated according to ISO 6976:2016, with HHV as the primary parameter for gas-quality assessment. HHV evaluation is performed in compliance with the OIML R 140 standard [45]. According to OIML R 140, Class A instruments must achieve a relative HHV accuracy within ±0.5% and a repeatability not exceeding 0.1% of the mean under identical conditions, while simultaneously satisfying the accuracy requirement.

### 3.3. Validation Procedure

The system was validated using representative NG and HNG mixtures (Table 2), including: NG1 (low methane, high heavy hydrocarbons, high HHV), NG2 (typical medium-quality NG), NG3 (methane-rich), HNG1–HNG2 (NG with 1% and 20% H_2_), and a binary CH_4_/H_2_ mixture (90/10). The balance (BAL) fraction groups minor species including heavier hydrocarbons and ppm-level traces of inert gases such as argon.

The instrument was validated over its operating range (−20 °C to 50 °C) using climatic chamber tests, with thermal ramps of 10 °C/h applied from −20 °C to 50 °C for each mixture. Validation tests were conducted at ~4 bara, representative of typical NG operating conditions, with additional pressures showing consistent results. Measurements were performed with gas samples under steady-state conditions, with additional dynamic flow tests confirming consistent performance.

## 4. Results and Discussion

**Figure 5:**
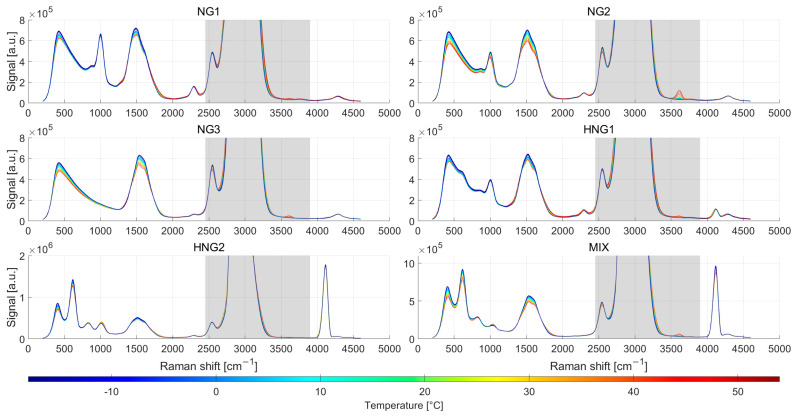
Experimental Raman spectra acquired during the thermal test campaigns performed inside the climatic chamber. Each spectrum was obtained by averaging 4 frames with 5 s integration time, corresponding to a total integration time of 20 s.

Figure 5 reports all the experimental spectra acquired with the instrument during the thermal campaigns. The data show that neither the spectra nor their overall morphology is significantly affected by variations in the instrument temperature or by the gas temperature inside the measurement cell. Notably, the spectral shape clearly reflects the composition of the analyzed mixture: in the NG1 spectra, the contribution of higher hydrocarbons, particularly ethane, is well distinguishable; in NG2 this contribution decreases; NG3 is almost entirely composed of methane and therefore exhibits a much simpler spectral pattern. The HNG1 and HNG2 spectra exhibit the Raman signature of hydrogen, while the MIX spectrum reflects its binary composition.

For NG2 and NG3, a weak water vapor feature at 3657 cm^−1^ can be observed at high temperatures. This signal is attributed to residual condensed water initially trapped in small gaps within the gas cell, such as flange interfaces or O-ring seats, which becomes released as the instrument is heated during the test campaigns. Importantly, this water peak lies outside the spectral region used in the fitting algorithm and therefore does not introduce crosstalk with the Raman bands employed for the compositional analysis.

All spectra were processed using the fitting routine to retrieve gas composition, from which thermodynamic parameters, including HHV, were calculated. The method provides a compositional resolution down to approximately 0.01%. To prevent false-positive detections, a conservative operational detection threshold of 500 ppm was adopted. This threshold was selected to be higher than the LOD estimated using the same approach reported in Ref. [46]. Camera thermal noise, particularly at elevated temperatures, contributes to rounding effects without compromising reliability.

**Figure 6:**
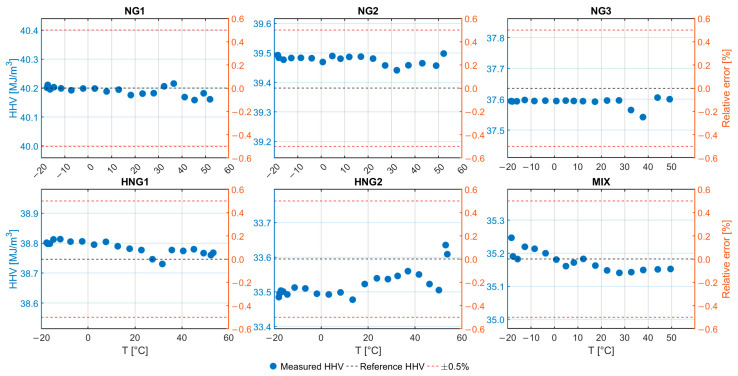
Measured HHVs for all tested mixtures (solid dots). The reference HHVs provided by the certified gas labels are indicated by the black dashed lines. The tolerance interval defining Class A according to the OIML R 140 specification is represented by the two red dashed lines.

The numerical results are reported in Appendix A. HHV results, reported in Figure 6, remain within OIML R 140 Class A limits, with errors below ±0.4% across the full temperature range, confirming the robustness of both calibration model and opto-mechanical design under thermal stress. Internal temperatures exceeded ambient limits by approximately 5 °C, due to heat dissipation within the explosion proof enclosure.

It is worth noting that, over such a wide operating temperature range, the HHV measurements remain within the maximum errors allowed for OIML Class A instruments; therefore, metrological compliance is maintained over the full operating range. The fact that all measured HHVs remain within the OIML R 140 Class A tolerance over the full validation range indicates that the combined uncertainty of the measurement procedure is compatible with the intended gas-quality application. The combined uncertainty of the measurement chain arises from the propagation of contributions such as the certified composition of the reference mixtures, pressure normalization during calibration, detector noise, residual laser-power fluctuations, temperature-dependent background variations, and fitting residuals associated with partially overlapping Raman bands. Repeatability was evaluated according to the metrics defined by the OIML R 140 standard and was always fulfilled for measurements performed under the same experimental conditions. Repeatability was verified by performing ten repeated measurements under a controlled experimental condition. The estimated HHV values remained within a 0.1% span around the mean calculated value, while also satisfying the required accuracy criterion.

Regarding the tested mixtures, the BAL fraction is representative of minor real-gas components, including heavier hydrocarbons and trace inert species. Although heavier hydrocarbons exhibit higher individual HHVs than methane, their very low concentration makes their contribution to the overall thermodynamic characterization negligible. This is supported by the measured HHVs, which remained within the required accuracy limits. High-hydrogen mixtures (HNG2 and MIX) represent a stringent test case due to the significantly lower HHV of H_2_ compared to CH_4_, which reduces the allowable absolute error. Despite this, the instrument maintains Class A performance, demonstrating reliable hydrogen quantification and accurate tracking of its impact on HHV.

The use of an uncooled CMOS detector reduces cost, power consumption, and system complexity, but increases thermal noise at elevated temperature. This effect mainly affects weak spectral features and is mitigated by dark-frame subtraction, averaging of multiple frames, photodiode-based laser-power normalization, and fitting over extended spectral regions rather than isolated peak maxima. The practical detection threshold of approximately 500 ppm should therefore be interpreted for the calibrated target species under the adopted acquisition conditions, not as a general trace-detection limit for arbitrary contaminants. The validation results over the full temperature range confirm that the remaining signal-to-noise ratio is sufficient for HHV determination within the OIML R 140 Class A requirement.

Overall, the results confirm the robustness of the calibration strategy, the stability of the spectral fitting routine, and the suitability of the system for real-time, in situ NG and HNG quality assessment under industrial environmental conditions.

The achieved performance should be interpreted in light of the intended application of the instrument. Unlike several previously reported Raman gas analyzers, which were mainly optimized for trace detection, maximum spectral resolution, or laboratory-grade compositional accuracy, the present system was designed as an industrial gas-quality analyzer for direct field deployment. Table 3 summarizes representative Raman-based gas-analysis systems reported in the literature and compares their main characteristics with those of the present work.

The broadband nature of the laser source directly affects the spectral resolution of the system. The recorded Raman spectra result from the convolution between the molecular Raman response and the instrumental function, which is dominated by the approximately 2 nm linewidth of the diode. Consequently, narrow spectral structures are broadened and closely spaced features may be merged. This behavior is clearly observed, for example, in the H_2_ vibrational Raman band and in the CO_2_ Fermi doublet, whose individual fine spectral features are not resolved with the present configuration.

This characteristic limits the capability of the instrument to detect trace species or minor components with strongly overlapping Raman bands when compared with systems based on narrow-linewidth lasers, cooled detectors, multi-pass cells, or cavity-enhanced configurations. However, this limitation is compatible with the intended application of the instrument. The system is designed for NG and HNG composition and gas-quality monitoring, where the target components are CH_4_, C_2_H_6_, C_3_H_8_, n-C_4_H_10_, i-C_4_H_10_, N_2_, CO_2_, and H_2_, and where the main output is the HHV calculated from the retrieved composition. Since HHV is mainly governed by the major components of the mixture, the reduced spectral resolution does not prevent compliance with the OIML R 140 Class A requirements demonstrated in this work.

Conversely, trace contaminants, sulfur compounds, odorant species used in NG distribution, or non-calibrated minor hydrocarbons are outside the current analytical scope of the instrument and would require dedicated calibration procedures, improved spectral resolution, enhanced optical sensitivity, or longer acquisition times.

Long-term on-site validation was performed in NG pressure-reduction stations under real operating conditions. The Raman system operated continuously over twelve months in a bypass configuration (~4 bara, ~1 L/min) and was periodically validated against certified reference gas mixtures, whose certified compositions were determined by gas chromatographic analysis performed by an accredited laboratory. The HHVs showed good agreement with the reference, with an average offset of ~0.3% and a maximum deviation below 0.42%. The temporal trends were consistent with the GC data, confirming system stability and repeatability. No significant drift or maintenance requirements were observed during operation, supporting the suitability of the instrument for long-term field deployment.

During field operation, the beam profile was not continuously monitored with a dedicated imaging system, since the instrument was installed in its final enclosed configuration. However, no evidence of beam-pointing or beam-profile instability affecting the measurements was observed. Optical stability was indirectly assessed through the photodiode signal, the overall Raman intensity, the fitting residuals, and the long-term stability of the calculated HHV. No systematic signal degradation or increase in fitting residuals was detected.

## 5. Conclusions

A Raman-based instrument for in-line measurement of NG and HNG composition in transportation and distribution networks has been developed. The system enables continuous and distributed assessment of gas quality in compliance with relevant metrological standards. It operates over −20 °C to 50 °C without carrier gases or sample manipulation, requiring only particulate filtration upstream and returning the sample to the network after measurement. The instrument covers pressures from 1.5 to 17 bara and employs an auto-adjusting integration-time algorithm, using a single calibration set acquired at ambient conditions.

The instrument has been metrologically validated by third-party certification bodies, demonstrating compliance with OIML R 140 Class A requirements over pressures from 1.5 to 17 bara and temperatures from −20 °C to 55 °C. Certification also includes mechanical class M1 and electromagnetic class E2, confirming suitability for direct deployment in NG infrastructures.

No uncontrolled drift or hysteresis effects were observed during thermal or field tests, confirming mechanical robustness and long-term reliability. Continuous operation over twelve months without maintenance further supports system stability. To the authors’ knowledge, this is the first implementation of a broadband laser diode for precision Raman spectroscopy of NG and HNG in an industrial application. The proposed system provides a practical solution for continuous gas-quality monitoring, enabling smart distribution and controlled blending strategies over a wide range of mixture compositions, including hydrogen-enriched mixtures.

The flexibility of the Raman technique further extends the potential applicability of the instrument to other gas mixtures, such as biogas and syngas, within the limits of the molecular species detectable by Raman spectroscopy. Extension to these matrices can be approached by applying the same calibration procedure used in this work to the missing major components. Biogas is mainly composed of CH_4_ and CO_2_, which are already included in the present calibration database, while additional species such as O_2_, which may be present up to approximately 2%, could be implemented by acquiring its Raman emission around 1555 cm^−1^. Similarly, syngas is mainly composed of H_2_ and CO, together with variable amounts of CO_2_, CH_4_, and N_2_. CO, which may reach concentrations up to approximately 50%, and would require an additional calibration spectrum. Its main Raman band around 2143 cm^−1^ and Raman cross section comparable to that of N_2_ make its inclusion compatible with the fitting approach adopted here. Therefore, the major components of these matrices can in principle be incorporated into the analysis by extending the calibration database. The limitations discussed for trace species remain valid, and contaminants or minor components would require dedicated calibration and validation. Future developments will focus on adapting both the system configuration and the spectral analysis algorithm to these matrices. In addition, the validated measurement cycle of 20 s per spectrum enables the instrument to sample composition changes at this rate. This temporal resolution is compatible with typical gas-blending processes, where composition changes are generally not instantaneous and, once the target blending condition is reached, the mixture is expected to remain sufficiently stable during operation.

## 6. Patents

This work is related to the patented device for gas analysis using Raman spectroscopy held by Pietro Fiorentini S.p.A. [41].

## Figures and Tables

**Table 1:** Molecular HHVs of the main gas components expressed on a molar and volumetric basis.

	HHV [MJ/mol]	HHV [MJ/m^3^] @ 15 °C, 1 atm
Methane, CH_4_	0.89	39.8
Ethane, C_2_H_6_	1.56	68.4
Propane, C_3_H_8_	2.22	93.0
n-Butane, nC_4_H_10_	2.87	121.0
i-Butane, iC_4_H_10_	2.86	120.5
Hydrogen, H_2_	0.286	12.7
Nitrogen, N_2_	–	–
Carbon dioxide, CO_2_	–	–

**Table 2:** Compositions of the tested mixtures. HHV calculated T1 = 15 °C, T2 = 15 °C.

	NG1	NG2	NG3	HNG1	HNG2	MIX
CH_4_	85.10	91.57	99.48	91.81	74.15	89.94
C_2_H_6_	9.00	5.05	0.04	4.12	3.32	0.00
C_3_H_8_	1.52	0.98	0.01	0.81	0.66	0.00
n-C_4_H_10_	0.21	0.15	0.00	0.14	0.11	0.00
i-C_4_H_10_	0.14	0.14	0.00	0.12	0.09	0.00
N_2_	2.47	0.99	0.40	1.28	1.03	0.00
CO_2_	1.43	1.03	0.05	0.65	0.52	0.00
H_2_	0.00	0.00	0.00	1.00	20.04	10.06
BAL	0.13	0.10	0.01	0.08	0.07	0.00
HHV [kJ/m^3^]	40,199.50	39,380.21	37,636.04	38,746.66	33,594.57	35,182.25

**Table 3:** Comparison between representative Raman-based gas-analysis systems reported in the literature and the system proposed in this work.

Refs.	Target Gases/Application	Optical Strategy	Acquisition Time	Operating Conditions	Main Performance	Deployment
[33,34]	NG composition analysis	532 nm narrow-linewidth, cooled CCD	Tens of seconds	Controlled laboratory conditions	Accurate NG composition, low detection limits for main species	Laboratory oriented, not specifically designed for field deployment
[35]	NG/CH_4_ detection	Cavity enhanced with optical feedback	Seconds—minutes	Laboratory cavity configuration	Enhanced Raman signal and improved sensitivity	High optical complexity; cavity alignment and stability requirements
[36]	Wobbe-index monitoring of NG	Holow-core fiber enhanced	Application– dependent	Controlled experimental setup	Demonstrated Wobbe-index monitoring	Promising for gas monitoring, not presented as legal-metrology compliant industrial instrumentation
[37]	CH_4_ detection	Cavity enhanced	60 s	Optimized controlled conditions	Enhanced methane sensitivity	Optimized for trace detection
This work	NG/HNG composition and HHV	450 nm broadband diode laser, custom high-throughput spectrometer, uncooled CMOS	20 s	Controlled validation/field operations	HHV measurements within OIML R 140 class A/12 month field stability	Designed for industrial field deployment and gas-quality monitoring

## Data Availability

The data supporting the conclusions of this article will be made available by the authors on request.

## Abbreviations

The following abbreviations are used in this manuscript:

ATEX	Atmosphères Explosibles
BAL	Balance
bara	Absolute bar
CMOS	Complementary Metal–Oxide–Semiconductor
DPSS	Diode-Pumped Solid-State
FTIR	Fourier Transform Infrared
GC	Gas Chromatography
HHV	Higher Heating Value
HNG	Hydrogen-Enriched Natural Gas
ISO	International Organization for Standardization
LNG	Liquefied Natural Gas
LOD	Limit of Detection
MEMS	Micro-Electro-Mechanical Systems
NG	Natural Gas
OIML	International Organization of Legal Metrology
TCD	Thermal Conductivity Detector
TDLAS	Tunable Diode Laser Absorption Spectroscopy
TEC	Thermo-Electric Cooler

## Appendix A

In this section, the numerical results of the thermal test campaigns performed on the instrument are reported. For each experimental temperature, the individual gas components and the corresponding HHV are provided.

**Table A1:** Measured compositions of NG1 mixture. HHV calculated T1 = 15 °C, T2 = 15 °C.

T [°C]	CH_4_ [%]	C_2_H_6_ [%]	C_3_H_8_ [%]	n-C_4_H_10_ [%]	i-C_4_H_10_ [%]	N_2_ [%]	CO_2_ [%]	H_2_ [%]	HHV [kJ/m^3^]
−18	84.98	9.00	1.24	0.62	0.16	2.64	1.37	0.00	40,201.3
−17	84.97	9.00	1.25	0.62	0.16	2.64	1.36	0.00	40,210.9
−16	85.02	8.97	1.24	0.63	0.15	2.63	1.37	0.00	40,195.7
−15	84.96	8.98	1.26	0.62	0.17	2.63	1.39	0.00	40,203.0
−12	85.03	8.96	1.24	0.69	0.09	2.62	1.36	0.00	40,199.0
−7	84.89	8.97	1.29	0.59	0.19	2.62	1.46	0.00	40,192.4
−2	84.94	8.96	1.30	0.61	0.15	2.59	1.44	0.00	40,198.5
3	84.96	8.96	1.28	0.66	0.11	2.60	1.43	0.00	40,198.4
8	84.89	8.97	1.32	0.60	0.15	2.60	1.48	0.00	40,188.4
13	84.93	8.97	1.30	0.66	0.10	2.59	1.45	0.00	40,194.5
18	84.86	8.98	1.32	0.59	0.15	2.60	1.50	0.00	40,175.8
23	84.85	8.99	1.33	0.61	0.13	2.60	1.50	0.00	40,180.8
28	84.85	9.00	1.33	0.59	0.13	2.58	1.50	0.00	40,182.3
32	84.94	9.02	1.31	0.65	0.08	2.52	1.48	0.00	40,206.1
37	85.05	9.04	1.28	0.72	0.00	2.47	1.45	0.00	40,215.6
41	84.96	9.01	1.31	0.59	0.11	2.55	1.47	0.00	40,168.8
45	84.94	9.07	1.29	0.59	0.09	2.58	1.44	0.00	40,158.6
49	85.02	9.09	1.21	0.72	0.00	2.59	1.36	0.00	40,182.0
52	85.02	9.09	1.23	0.63	0.06	2.60	1.37	0.00	40,161.2

**Table A2:** Measured compositions of NG2 mixture. HHV calculated T1 = 15 °C, T2 = 15 °C.

T [°C]	CH_4_ [%]	C_2_H_6_ [%]	C_3_H_8_ [%]	n-C_4_H_10_ [%]	i-C_4_H_10_ [%]	N_2_ [%]	CO_2_ [%]	H_2_ [%]	HHV [kJ/m^3^]
−18	91.49	5.20	0.83	0.48	0.09	0.99	0.93	0.00	39,492.2
−18	91.46	5.20	0.83	0.48	0.08	1.00	0.94	0.00	39,483.4
−16	91.45	5.21	0.85	0.46	0.09	0.99	0.95	0.00	39,476.7
−13	91.39	5.21	0.86	0.47	0.09	1.01	0.97	0.00	39,482.7
−9	91.30	5.22	0.89	0.43	0.13	1.00	1.03	0.00	39,483.2
−4	91.29	5.23	0.90	0.43	0.12	0.99	1.04	0.00	39,481.8
1	91.27	5.21	0.90	0.44	0.12	0.99	1.07	0.00	39,469.0
5	91.28	5.22	0.90	0.45	0.11	0.99	1.05	0.00	39,489.4
8	91.16	5.24	0.94	0.39	0.16	1.00	1.11	0.00	39,480.3
12	91.16	5.24	0.93	0.41	0.15	1.00	1.11	0.00	39,486.4
17	91.17	5.24	0.93	0.41	0.15	1.00	1.10	0.00	39,487.2
22	91.08	5.27	0.95	0.38	0.17	1.01	1.13	0.00	39,480.5
27	91.12	5.28	0.90	0.45	0.11	1.06	1.09	0.00	39,457.4
32	91.05	5.29	0.89	0.45	0.11	1.10	1.10	0.00	39,441.1
37	91.15	5.29	0.86	0.50	0.07	1.02	1.11	0.00	39,457.9
43	91.13	5.30	0.91	0.41	0.13	1.01	1.11	0.00	39,464.8
49	91.12	5.32	0.88	0.42	0.12	1.03	1.10	0.00	39,456.7
52	91.06	5.36	0.88	0.41	0.16	1.02	1.11	0.00	39,497.7

**Table A3:** Measured compositions of NG3 mixture. HHV calculated T1 = 15 °C, T2 = 15 °C.

T [°C]	CH_4_ [%]	C_2_H_6_ [%]	C_3_H_8_ [%]	n-C_4_H_10_ [%]	i-C_4_H_10_ [%]	N_2_ [%]	CO_2_ [%]	H_2_ [%]	HHV [kJ/m^3^]
−18	99.51	0.00	0.00	0.00	0.00	0.49	0.00	0.00	37,594.3
−18	99.51	0.00	0.00	0.00	0.00	0.49	0.00	0.00	37,593.2
−16	99.51	0.00	0.00	0.00	0.00	0.49	0.00	0.00	37,593.8
−13	99.52	0.00	0.00	0.00	0.00	0.48	0.00	0.00	37,597.9
−9	99.51	0.00	0.00	0.00	0.00	0.49	0.00	0.00	37,594.6
−4	99.52	0.00	0.00	0.00	0.00	0.48	0.00	0.00	37,595.7
1	99.51	0.00	0.00	0.00	0.00	0.49	0.00	0.00	37,594.7
5	99.52	0.00	0.00	0.00	0.00	0.48	0.00	0.00	37,595.9
8	99.51	0.00	0.00	0.00	0.00	0.49	0.00	0.00	37,594.8
12	99.51	0.00	0.00	0.00	0.00	0.49	0.00	0.00	37,594.1
17	99.51	0.00	0.00	0.00	0.00	0.49	0.00	0.00	37,592.5
22	99.52	0.00	0.00	0.00	0.00	0.48	0.00	0.00	37,596.1
28	99.52	0.00	0.00	0.00	0.00	0.48	0.00	0.00	37,596.5
33	99.44	0.00	0.00	0.00	0.00	0.51	0.05	0.00	37,565.3
38	99.37	0.00	0.00	0.00	0.00	0.51	0.11	0.00	37,542.5
44	99.24	0.06	0.00	0.06	0.00	0.50	0.14	0.00	37,605.6
50	99.17	0.07	0.00	0.07	0.00	0.50	0.19	0.00	37,600.5

**Table A4:** Measured compositions of HNG1 mixture. HHV calculated T1 = 15 °C, T2 = 15 °C.

T [°C]	CH_4_ [%]	C_2_H_6_ [%]	C_3_H_8_ [%]	n-C_4_H_10_ [%]	i-C_4_H_10_ [%]	N_2_ [%]	CO_2_ [%]	H_2_ [%]	HHV [kJ/m^3^]
−18	92.32	4.02	0.63	0.44	0.00	1.26	0.35	0.96	38,801.4
−17	92.30	4.03	0.63	0.45	0.00	1.26	0.37	0.96	38,797.8
−16	92.31	4.02	0.64	0.44	0.00	1.25	0.38	0.96	38,798.1
−15	92.24	4.02	0.67	0.39	0.05	1.26	0.40	0.97	38,812.4
−12	92.18	4.03	0.68	0.39	0.07	1.25	0.44	0.96	38,813.2
−8	92.03	4.05	0.73	0.33	0.11	1.25	0.53	0.97	38,805.0
−3	92.03	4.05	0.71	0.38	0.08	1.24	0.53	0.98	38,805.8
3	92.02	4.04	0.71	0.40	0.06	1.24	0.54	0.98	38,794.9
8	91.94	4.06	0.72	0.40	0.08	1.23	0.58	0.99	38,804.3
13	91.88	4.07	0.73	0.38	0.09	1.24	0.62	1.00	38,789.6
18	91.76	4.08	0.76	0.34	0.13	1.25	0.68	1.01	38,781.3
23	91.61	4.10	0.78	0.33	0.15	1.28	0.72	1.02	38,776.9
27	91.54	4.11	0.77	0.36	0.11	1.34	0.71	1.05	38,746.3
32	91.44	4.14	0.74	0.39	0.10	1.37	0.72	1.09	38,730.5
36	91.50	4.18	0.71	0.45	0.07	1.29	0.72	1.08	38,777.0
41	91.62	4.17	0.64	0.54	0.00	1.26	0.72	1.05	38,774.0
45	91.40	4.20	0.73	0.38	0.15	1.28	0.79	1.07	38,779.6
49	91.41	4.20	0.71	0.38	0.15	1.30	0.78	1.07	38,766.6
52	91.51	4.20	0.69	0.40	0.11	1.28	0.74	1.08	38,760.1
53	91.44	4.20	0.70	0.36	0.17	1.30	0.75	1.08	38,767.9

**Table A5:** Measured compositions of HNG2 mixture. HHV calculated T1 = 15 °C, T2 = 15 °C.

T [°C]	CH_4_ [%]	C_2_H_6_ [%]	C_3_H_8_ [%]	n-C_4_H_10_ [%]	i-C_4_H_10_ [%]	N_2_ [%]	CO_2_ [%]	H_2_ [%]	HHV [kJ/m^3^]
−18	74.32	3.08	0.65	0.00	0.27	0.97	0.50	20.21	33,484.7
−18	74.33	3.09	0.65	0.00	0.28	0.97	0.50	20.20	33,495.1
−17	74.28	3.09	0.66	0.00	0.28	0.97	0.50	20.22	33,503.5
−16	74.26	3.10	0.65	0.00	0.28	0.96	0.51	20.22	33,500.8
−14	74.19	3.11	0.66	0.00	0.28	0.97	0.52	20.26	33,492.5
−11	74.03	3.15	0.66	0.00	0.33	0.96	0.56	20.31	33,512.3
−7	73.86	3.17	0.68	0.00	0.34	0.95	0.58	20.42	33,509.8
−2	73.70	3.20	0.71	0.00	0.33	0.95	0.58	20.54	33,494.5
3	73.48	3.24	0.69	0.00	0.38	0.95	0.63	20.64	33,492.4
8	73.26	3.30	0.68	0.00	0.42	0.96	0.66	20.72	33,498.3
13	73.16	3.31	0.72	0.00	0.38	0.95	0.63	20.84	33,477.3
19	72.89	3.40	0.68	0.00	0.48	0.95	0.70	20.89	33,522.5
24	72.80	3.45	0.68	0.00	0.49	0.95	0.71	20.92	33,539.1
28	72.71	3.45	0.56	0.25	0.35	1.00	0.70	20.97	33,536.7
33	72.48	3.53	0.56	0.24	0.40	1.07	0.72	21.00	33,545.9
37	72.47	3.55	0.47	0.38	0.33	1.04	0.72	21.04	33,559.8
42	72.29	3.60	0.38	0.51	0.27	1.00	0.72	21.23	33,550.1
46	72.16	3.59	0.29	0.71	0.14	1.01	0.67	21.43	33,522.5
50	72.05	3.62	0.23	0.81	0.07	1.03	0.67	21.51	33,505.0
53	72.34	3.65	0.21	0.93	0.00	1.02	0.63	21.21	33,635.2
54	72.23	3.70	0.18	0.94	0.00	1.04	0.64	21.27	33,608.8

**Table A6:** Measured compositions of MIX mixture. HHV calculated T1 = 15 °C, T2 = 15 °C.

T [°C]	CH_4_ [%]	C_2_H_6_ [%]	C_3_H_8_ [%]	n-C_4_H_10_ [%]	i-C_4_H_10_ [%]	N_2_ [%]	CO_2_ [%]	H_2_ [%]	HHV [kJ/m^3^]
−18	89.93	0.00	0.00	0.00	0.07	0.06	0.00	9.94	35,246.9
−18	90.00	0.00	0.00	0.00	0.00	0.05	0.00	9.95	35,190.5
−16	89.97	0.00	0.00	0.00	0.00	0.06	0.00	9.98	35,182.4
−13	89.85	0.00	0.00	0.00	0.06	0.05	0.00	10.04	35,219.6
−8	89.76	0.00	0.00	0.00	0.08	0.05	0.00	10.11	35,213.6
−4	89.67	0.00	0.00	0.00	0.09	0.05	0.00	10.19	35,200.0
1	89.61	0.00	0.00	0.00	0.08	0.06	0.00	10.25	35,180.9
5	89.54	0.00	0.00	0.00	0.08	0.06	0.00	10.32	35,161.0
8	89.53	0.00	0.00	0.00	0.09	0.00	0.00	10.38	35,171.8
12	89.42	0.00	0.00	0.00	0.12	0.00	0.00	10.46	35,182.9
18	89.33	0.00	0.00	0.00	0.13	0.00	0.00	10.55	35,162.7
23	89.21	0.00	0.00	0.05	0.08	0.00	0.00	10.65	35,148.1
28	89.11	0.00	0.00	0.06	0.09	0.00	0.00	10.73	35,140.7
33	88.97	0.08	0.00	0.15	0.00	0.00	0.00	10.80	35,142.7
38	88.76	0.14	0.00	0.19	0.00	0.00	0.11	10.80	35,149.3
44	88.62	0.16	0.00	0.22	0.00	0.00	0.15	10.86	35,151.2
50	88.42	0.22	0.00	0.24	0.00	0.00	0.17	10.96	35,152.5

## References

[1] Adenubi S., Appah D., Okafor E., Aimikhe V. (2023). A Review of Leak Detection Systems for Natural Gas Pipelines and Facilities. Int. J. Energy Technol. Policy.

[2] Zhang S., Xie S., Li Y., Yuan M., Qian X. (2023). Detection of Gas Pipeline Leakage Using Distributed Optical Fiber Sensors: Multi-Physics Analysis of Leakage-Fiber Coupling Mechanism in Soil Environment. Sensors.

[3] ENTSOG (2024). Gas Quality Monitoring Report—First Edition.

[4] Keogh N., Corr D., Monaghan R.F.D. (2022). Biogenic renewable gas injection into natural gas grids: A review of technical and economic modelling studies. Renew. Sustain. Energy Rev..

[5] Quintino F.M., Nascimento N., Fernandes E.C. (2021). Aspects of hydrogen and biomethane introduction in natural gas infrastructure and equipment. Hydrogen.

[6] Mahajan D., Tan K., Venkatesh T., Kileti P., Clayton C.R. (2022). Hydrogen blending in gas pipeline networks—A review. Energies.

[7] Bloj M.-D., Ripeanu R.G., Diniță A., Oprea V.O., Tănase M. (2025). Comprehensive review of hydrogen-natural gas blending: Global project insights with a focus on implementation and impact in Romanian gas networks. Heliyon.

[8] Vaccariello E., Trinchero R., Stievano I.S., Leone P. (2021). A statistical assessment of blending hydrogen into gas networks. Energies.

[9] (2016). Natural Gas—Calculation of Calorific Values, Density, Relative Density and Wobbe Index from Composition.

[10] Brown A.S., Milton M.J.T., Cowper C.J., Squire G.D., Bremser W., Branch R.W. (2004). Analysis of natural gas by gas chromatography: Reduction of correlated uncertainties by normalisation. J. Chromatogr. A.

[11] Ficco G., Cigolotti V., Cortellessa G., Monteleone G., Dell’Isola M. (2025). Impact of hydrogen-enriched natural gas on the accuracy of odorant measurements. Sensors.

[12] Regmi B.P., Agah M. (2018). Micro gas chromatography: An overview of critical components and their integration. Anal. Chem..

[13] Emerson Fundamentals of Gas Chromatography.

[14] (2012). Natural Gas—Determination of Composition and Associated Uncertainty by Gas Chromatography.

[15] Crucello J., Oliveira A.M., Sampaio N.M.F.M., Hantao L.W. (2022). Miniaturized systems for gas chromatography: Developments in sample preparation and instrumentation. J. Chromatogr. A.

[16] Agilent Technologies (2023). Natural Gas Analyzer GC Systems.

[17] Singh R., Gupta R., Bansal D., Bhateria R., Sharma M. (2024). A review on recent trends and future developments in electrochemical sensing. ACS Omega.

[18] Laref R., Losson E., Sava A., Siadat M. (2021). Empiric unsupervised drifts correction method of electrochemical sensors for in-field nitrogen dioxide monitoring. Sensors.

[19] Rivera P., Della Pelle F., Stonyte J., Martins Antunes de Melo W.C., Abouhagger A., Pauliukaite R. (2025). Overcoming challenges in electrochemical sensing: Toward continuous monitoring. ACS Sens..

[20] Ba Hashwan S.S., Khir M.H.M., Nawi I.M., Ahmad M.R., Hanif M., Zahoor F., Al-Douri Y., Algamili A.S., Bature U.I., Alabsi S.S. (2023). A review of piezoelectric MEMS sensors and actuators for gas detection application. Discov. Nano.

[21] Wu Y., Lei M., Xia X. (2024). Research progress of MEMS gas sensors: A comprehensive review of sensing materials. Sensors.

[22] Luo W., Dai F., Liu Y., Wang X., Li M. (2025). Pulse-driven MEMS gas sensor combined with machine learning for selective gas identification. Microsyst. Nanoeng..

[23] Hamaguchi R. (2018). Natural gas measurement using temperature-dependent thermal conductivity. Tech. Rev..

[24] Avetisov V., Bjoroey O., Wang J., Geiser P., Paulsen K.G. (2019). Hydrogen sensor based on tunable diode laser absorption spectroscopy. Sensors.

[25] Liang T., Qiao S., Liu X., Ma Y. (2022). Highly sensitive hydrogen sensing based on tunable diode laser absorption spectroscopy with a 2.1 μm diode laser. Chemosensors.

[26] Nie Q., Wang Z., Ren W. (2025). Cavity-enhanced absorption spectroscopy with continuous and fast wavelength tuning. Chin. Opt. Lett..

[27] Chu L.-K., Huang Y.-H., Lee Y.-P., Gupta V.P. (2022). Step-scan FTIR techniques for investigations of spectra and dynamics of transient species in gaseous chemical reactions. Molecular and Laser Spectroscopy.

[28] Long D.A. (2002). The Raman Effect—A Unified Treatment of the Theory of Raman Scattering by Molecules.

[29] Hollas J.M. (2003). Modern Spectroscopy.

[30] Popp J., Mayerhöfer T. (2020). Micro-Raman Spectroscopy: Theory and Application.

[31] Huang B., Zhao Q., Sun C., Zhu L., Zhang H., Zhang Y., Liu C., Li F. (2022). Trace analysis of gases and liquids with spontaneous Raman scattering based on the integrating sphere principle. Anal. Chem..

[32] Danichkin S.A., Eliseev A.A., Popova T.N., Ravodina O.V., Stenina V.V. (1981). Raman scattering parameters for gas molecules (survey). J. Appl. Spectrosc..

[33] Buldakov M.A., Korolkov V.A., Matrosov I.I., Petrov D.V., Tikhomirov A.A., Korolev B.V. (2013). Analyzing natural gas by spontaneous Raman scattering spectroscopy. J. Opt. Technol..

[34] Petrov D.V., Matrosov I.I. (2016). Raman gas analyzer (RGA): Natural gas measurements. Appl. Spectrosc..

[35] Hippler M. (2015). Cavity-enhanced Raman spectroscopy of natural gas with optical feedback cw-diode lasers. Anal. Chem..

[36] Sandfort V., Trabold B.M., Abdolvand A., Bolwien C., Russell P.S.J., Wöllenstein J., Palzer S. (2017). Monitoring the Wobbe index of natural gas using fiber-enhanced Raman spectroscopy. Sensors.

[37] Qin Y., Sun X., Shi G., Ning R., Li X., Shen L. (2026). Methane gas detection based on cavity-enhanced Raman spectroscopy. J. Phys. Conf. Ser..

[38] Dal Moro R., Melison F., Cocola L., Poletto L. (2026). Raman Spectroscopy for Monitoring NOx and N2O in Combustion Products. Sensors.

[39] Cocola L., Melison F., Scarabottolo N., Tondello G., Poletto L. Diode-based Raman sensor for fuel gas analysis. Proceedings of the SPIE—Optical Sensing and Detection VI.

[40] Melison F., Cocola L., Meneghin E., Rossi D., Poletto L. Raman spectroscopy applied to in-situ natural gas and hydrogen-enriched natural gas composition measurements. Proceedings of the SPIE—Next-Generation Spectroscopic Technologies XVII.

[41] Pietro Fiorentini S.p.A. (2024). Device for Gas Analysis Using Raman Spectroscopy. European Patent.

[42] (2026). Explosive Atmospheres—All Parts, Including IEC 60079-0, Explosive Atmospheres—Part 0: Equipment—General Requirements, and IEC 60079-1, Explosive Atmospheres—Part 1: Equipment Protection by Flameproof Enclosures “d”.

[43] Riedl M.J. (2001). Optical Design Fundamentals for Infrared Systems.

[44] Qi J., Bechtel K.L., Shih W.-C. (2014). Automated image curvature assessment and correction for high-throughput Raman spectroscopy and microscopy. Biomed. Spectrosc. Imaging.

[45] (2007). Measuring Systems for Gaseous Fuels.

[46] Melison F., Cocola L., Poletto L. (2025). Raman gas sensor for hydrogen detection via non-dispersive and dispersive approaches. Sensors.

